# Growth and Bioactive Compound Content of *Glehnia littoralis* Fr. Schmidt ex Miquel Grown under Different CO_2_ Concentrations and Light Intensities

**DOI:** 10.3390/plants9111581

**Published:** 2020-11-15

**Authors:** Hye Ri Lee, Hyeon Min Kim, Hyeon Woo Jeong, Myung Min Oh, Seung Jae Hwang

**Affiliations:** 1 Division of Applied Life Science, Graduate School of Gyeongsang National University, Jinju 52828, Korea; dgpfl77@naver.com (H.R.L.); hmk0766@korea.kr (H.M.K.); J_dk94@naver.com (H.W.J.); 2 Division of Animal, Horticulture and Food Sciences, Chungbuk National University, Cheongju 28644, Korea; moh@cbnu.ac.kr; 3 Department of Agricultural Plant Science, College of Agriculture & Life Science, Gyeongsang National University, Jinju 52828, Korea; 4 Institute of Agriculture & Life Science, Gyeongsang National University, Jinju 52828, Korea; 5 Research of Institute of Life Science, Gyeongsang National University, Jinju 52828, Korea

**Keywords:** chlorogenic acid, medicinal plant, photosynthetic rate, total saponin

## Abstract

This study aims to determine the effect of different CO_2_ concentrations and light intensities on the growth, photosynthetic rate, and bioactive compound content of *Glehnia littoralis* Fr. Schmidt ex Miquel in a closed-type plant production system (CPPS). The plants were transplanted into a deep floating technique system with recycling nutrient solution (EC 1.0 dS·m^-1^ and pH 6.5) and cultured for 96 days under a temperature of 20 ± 1 °C, a photoperiod of 12/12 h (light/dark), and RGB LEDs (red:green:blue = 7:1:2) in a CPPS. The experimental treatments were set to 500 or 1500 µmol∙mol^−1^ CO_2_ concentrations in combination with one of the three light intensities: 100, 200, or 300 µmol∙m^−2^∙s^−1^ photosynthetic photon flux density (PPFD). The petiole length of *G. littoralis* was the longest in the 500 µmol∙mol^−1^ CO_2_ concentration with the 100 µmol∙m^−2^∙s^−1^ PPFD. The fresh weight (FW) and dry weight (DW) of shoots and roots were the heaviest in the 300 µmol∙m^−2^∙s^−1^ PPFD regardless of the CO_2_ concentration. Higher CO_2_ concentrations and light intensities produced the greatest photosynthetic rates. However, the SPAD value was not significantly different between the treatments. Higher light intensities produced greater content per biomass of chlorogenic acid and total saponin, although the concentration per DW or FW was not significantly different between treatments. The first and second harvest yields were the greatest in the 300 µmol∙m^−2^∙s^−1^ PPFD, regardless of the CO_2_ concentration. These results show that the 300 µmol∙m^−2^∙s^−1^ PPFD enhanced the growth, photosynthetic rate, and bioactive compound accumulation of *G. littoralis*, regardless of the CO_2_ concentration in a CPPS.

## 1. Introduction

Medicinal plants are those plants where the whole leaves, stems, flowers, fruits, roots, seeds, or whole plants are used as raw materials for medicine. Currently, medicinal plants are being used in various ways as herbal medicines and as the raw materials of cosmetics, functional health foods, and natural medicines; the interest in these products from consumers has increased, thus increasing the demand and related market size [1]. In the Republic of Korea, various medicinal plants are native in the wild because of Korea’s geographical characteristics and the four distinct seasons. However, only about 50 kinds of medicinal plants are grown in domestic farms, and there are fewer cultivation areas and productions than can supply the increasing demand; therefore, the dependence on imports is also increasing [2]. Because medicinal plants are mainly grown outdoors, the cultivation period is long, the price fluctuation is severe, and the supply of medicinal plants is inelastic. In addition, the harvest time is limited; they are difficult to store or transport; the quality varies between varieties, cultivation environment, and harvest time. Also, the study of the cultivation process for the mass production of medicinal plants (seed germination, growing seedlings, cultivation after transplanting, etc.,) is insufficient, and it is necessary to find suitable conditions for the cultivation environment and develop the optimal cultivation technology for the stable production of high-quality medicinal plants. 

*Glehania littoralis* Fr. Schmidt ex Miquiel is a perennial plant belonging to the Umbelliferae family and is commonly found in coastal dunes. The petiole is long and scarlet, and the leaves are divided into three branches, each having three small leaves, where the small leaves have sawtooth edges, and the young leaves are used as vegetables. It has several functional components, such as saponin, coumarin, bergapten, β-sitosterol, and imperatorin [3,4], which are known to be effective against sweating, fever, and labor pains, and have diuretic, antiviral, anti-cancer, and immunosuppression properties [5,6,7].

CO_2_ is a very important factor in crop cultivation as a raw material for photosynthesis. The maintenance of CO_2_ concentrations during growth enhances crop growth [8,9]. CO_2_ enrichment can enhance CO_2_ fixation, yield, and quality [10,11]. Previous results showed that the biomass increased by about 50% in C3 plants [12], 35% in CAM (crassulacean acid metabolism) plants [13], and 12% in C4 plants [14] under CO_2_ enrichment conditions. Moreover, CO_2_ enrichment is effective in enhancing vegetable quality by promoting the accumulation of antioxidants in vegetables. Dong et al. [15] showed that CO_2_ enrichment increased the ascorbic acid, chlorophyll b, total antioxidant activity, total phenols, and total flavonoids by 9.5, 42.5, 59.0, 8.9, and 45.5%, respectively. However, the studies on CO_2_ enrichment in medicinal plants are relatively limited compared to those on the other plants.

Light is an energy source for plant photosynthesis and an essential for plant growth, development, and bioactive compound accumulation. To achieve significant levels of plant cultivation in artificial facilities, it is important to set appropriate environmental conditions, especially with regard to light intensity. Plants grown under low light intensity have frequently been found to be more susceptible to photoinhibition than in high light intensity [16]. Previous studies have shown a decrease in the photosynthesis of eggplant [17] and reduced dry weight of wheat [18] under the weak light conditions. The long-term weak lighting conditions resulted in smaller leaf areas and thinner leaves [19]. On the other hand, too high light intensity also has negative effect on plant growth. This induces leaf wilting and reduces leaf area, chlorophyll content, and photosynthesis efficiency [20]. It can also destroy photosynthetic systems and cause serious oxidative damage to leaf tissue [21].

Therefore, the objective of this study is to investigate the growth, photosynthesis, and bioactive compound content of medicinal plants, and consequently, find a suitable combination of CO_2_ concentration and light intensity for high-quality mass production in a closed-type plant production system (CPPS).

## 2. Results and Discussion

### 2.1. Growth Characteristics

**Figure 1:**
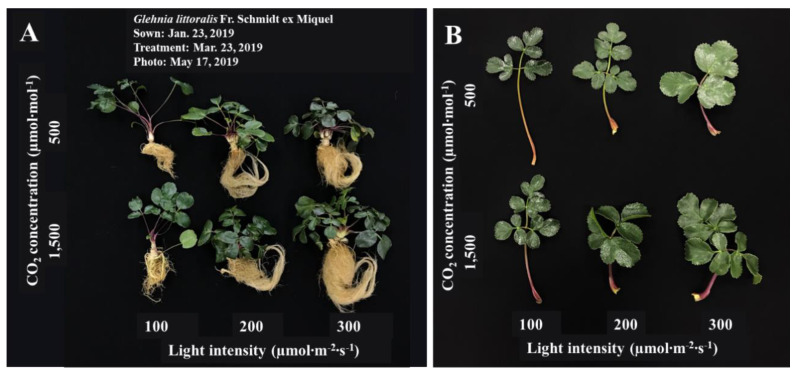
Images of the growth of the whole plant (**A**) and leaf (**B**) of *Glehnia littoralis* Fr. Schmidt ex Miquel as affected by different CO_2_ concentrations and light intensities.

The different shoot growth characteristics of *G. littoralis*, as affected by various CO_2_ concentrations and light intensities after 56 days of treatment, are shown in Figure 1 and Table 1. The petiole length was the longest at 8.8 cm in the 500 µmol∙mol^−1^ CO_2_ concentration with 100 µmol∙m^−2^∙s^−1^ PPFD, while the leaf area was the widest at 142.78 cm^2^ in the 500 µmol∙mol^−1^ with 300 PPFD (Figure 1B and Table 1). The crown diameter, number of leaves, fresh weight (FW), and dry weight (DW) of the shoots were the lowest in the 500 µmol∙mol^−1^ with 100 µmol∙m^−2^∙s^−1^ PPFD. The growth of shoots was more affected by the light intensity than by the CO_2_ concentration, and the growth of *G. littoralis* in higher light intensity (300 µmol∙m^−2^∙s^−1^ PPFD) was higher compared lower light intensity (100 µmol∙m^−2^∙s^−1^ PPFD). An appropriate light intensity is a major factor for growth, morphogenesis, and other physiological responses [22,23]. Plants grown in a low light intensity have frequently been shown to display more photoinhibition than those grown under a high light intensity [24]. A low light intensity condition may lead to stretching of leaf length, leaf width, and plant height [25]. The *G. littoralis* showed an insignificant difference in the specific leaf area (SLA) (data are not shown) but the petiole length was stretched.

The root growth characteristics, such as the root length, root diameter, FW, and DW of the roots are shown in Table 2. The root growth was closely associated with the light condition for the shoot growth [26]. The root length was the shortest in the 100 µmol∙m^−2^∙s^−1^ PPFD, regardless of the CO_2_ concentration, at 10.7 and 11.3 cm for the 500 and **1**500 µmol∙mol^−1^ concentration, respectively. The root diameter was the thinnest at 6.50 mm in the **1**500 µmol∙mol^−1^ with 100 µmol∙m^−2^∙s^−1^ PPFD. Furthermore, the FW and DW of roots displayed a positive correlation in which the weight increased as the light intensity increased. The root growth was not affected by the CO_2_ concentration but, a change in the light intensity produced a significant difference. Nager et al. [27] reported that a high light intensity (300 µmol∙m^−2^∙s^−1^ PPFD) enhanced the FW of the roots of *Nicotiana tabacum*. Similarly, Olschowski et al. [28] obtained a heavier root DW of *Calibrachoa* in a high light intensity when cutting. Kitaya et al. [29] suggested that an optimal PPFD can rapidly produce high-quality lettuce plug seedlings. 

### 2.2. Photosynthetic Rate and the SPAD Value 

**Figure 2:**
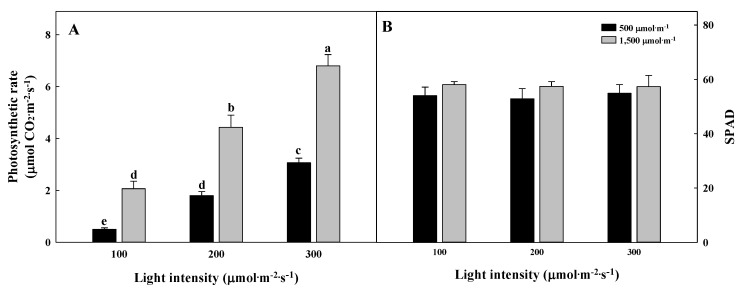
Photosynthetic rate (**A**) and SPAD (**B**) of *Glehnia littoralis* Fr. Schmidt ex Miquel, as affected by various CO_2_ concentrations and light intensities 56 days of treatment. The vertical bars represent the standard deviation of the mean (*n* = 3). Different letters above bars indicate significant differences at *p* ≤ 0.05, using Tukey’s multiple range test.

The CO_2_ concentration and light intensity significantly affected the photosynthetic rate in *G. littoralis* (Figure 2A). The photosynthetic rate was the highest in the 1500 µmol∙mol^−1^ CO_2_ concentration with the 300 µmol∙m^−2^∙s^−1^ PPFD in 6.8 µmol CO_2_ m^−2^∙s^−1^, while the lowest was found in the 500 µmol∙mol^-1^ CO_2_ concentration with 100 µmol∙m^−2^∙s^−1^ PPFD in 0.5 µmol CO_2_ m^−2^∙s^−1^. The photosynthetic rate showed a positive correlation with CO_2_ concentration and the light intensity. The SPAD did not show a significant difference (Figure 2B). Zheng et al. [30] reported that a high light intensity (350 µmol∙m^−2^∙s^−1^ PPFD) increased the photosynthetic capacity of the mother plant and the primary runner plant of strawberry. Furthermore, CO_2_ enrichment increased the photosynthetic rate of *Gerbera jamesonii* [31]. *G. littoralis* is a crop native to coastal dunes and grows in high-light natural environments. Similar results showed that the growth of *Peucedanum japonicum* Thunberg, which is native to the seashore, was also efficient at increasing growth and production in a high light intensity (200 µmol∙m^−2^∙s^−1^ PPFD) than in a low light intensity (60 µmol∙m^−2^∙s^−1^ PPFD) [32].

### 2.3. Total Sugar and Starch

**Figure 3:**
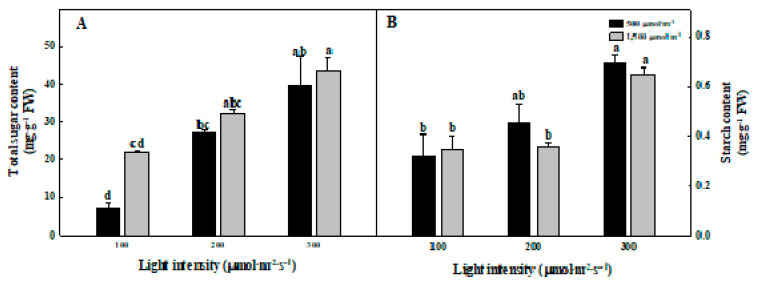
The different total sugar (**A**) and starch (**B**) contents of *Glehnia littoralis* Fr. Schmidt ex Miquel, as affected by various CO_2_ concentrations and light intensities after 56 days of treatment. The vertical bars represent the standard deviation of the mean (*n* = 3). Different letters above bars indicate significant differences at *p* ≤ 0.05, using Tukey’s multiple range test.

The high light intensity increased the total sugar and starch contents in *G. littoralis*. However, the CO_2_ concentration did not influence the total sugar and starch contents. The total sugar and starch contents were the highest in the 300 µmol∙m^−2^∙s^−1^ PPFD regardless of the CO_2_ concentration (Figure 3). In a CO_2_ concentration of 500 µmol∙mol^−1^, the total sugar and starch contents were higher in the 300 µmol∙m^−2^∙s^−1^ PPFD by 5.4 and 2.2 times compared to the 100 µmol∙m^−2^∙s^−1^ PPFD, respectively. Many studies have reported that CO_2_ enrichment increases the sugar and starch contents [33,34,35], which is inconsistent with this study. *G. littoralis*, a halophyte with developed water storage tissue that stores a lot of water in the cell, is considered to be less sensitive to CO_2_ concentrations because of its thick leaves. The leaf is a photosynthetic organ, and the area and thickness are the major factors affecting the growth of the plant. To absorb sufficient light energy, the leaves are as wide as possible, and at the same time to facilitate gas exchange (CO_2_, O_2_, and H_2_O), the leaves are as flat and thin as possible [36]. The higher light intensity can positively affect the accumulation of assimilates, such as proteins, and the starch, and sugar of spinach increased in the 300 µmol∙m^−2^∙s^−1^ PPFD than those in the 100 µmol∙m^−2^∙s^−1^ PPFD [37]. In *Glycine max* (Linn.) Merr., a high light intensity induced photosynthetic activity, increasing the soluble sugar, sucrose, and starch contents in the shoots and roots [38].

### 2.4. Harvest Yield 

**Figure 4:**
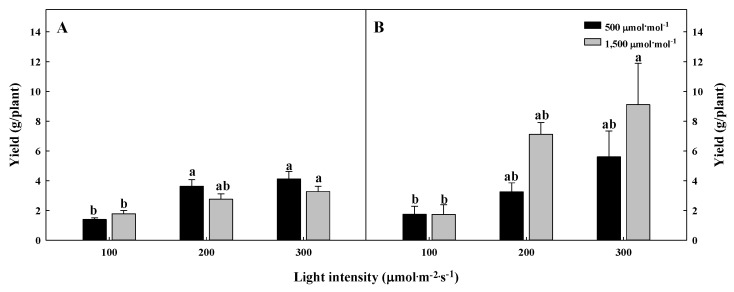
The different first (**A**) and second (**B**) harvest yields of *Glehnia littoralis* Fr. Schmidt ex Miquel, as affected by various CO_2_ concentrations and light intensities after 56 days of treatment. The vertical bars represent the standard deviation of the mean (*n* = 3). Different letters above bars indicate significant differences at *p* ≤ 0.05, using Tukey’s multiple range test.

**Figure 5:**
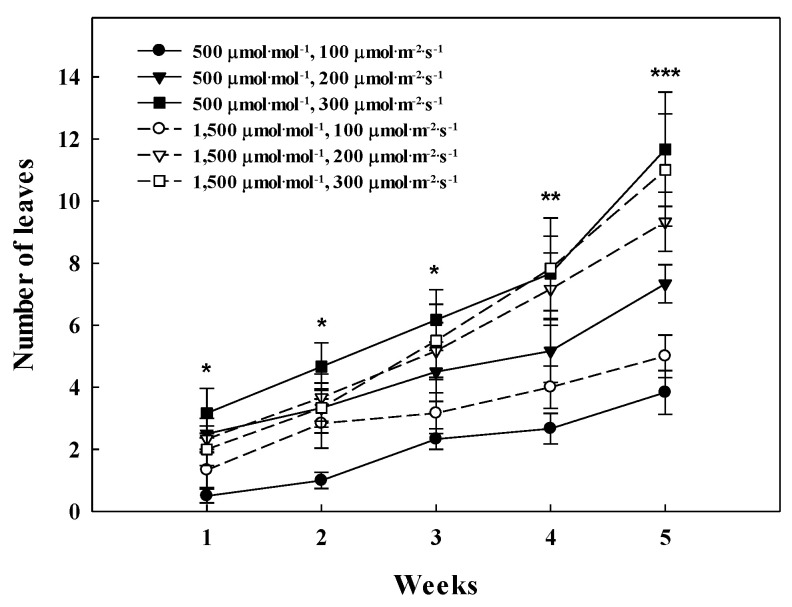
Changes in the number of leaves on *Glehnia littoralis* Fr. Schmidt ex Miquel, as affected by various CO_2_ concentrations and light intensities. The error bars represent the standard deviation of the mean (*n* = 3). *, **, *** Significant at *p* ≤ 0.05, 0.01, or 0.001, respectively.

*G. littoralis* can be harvested cyclically by taking a leaf. The CO_2_ enrichment did not have a significant effect on the harvest yield or the change in the number of leaves from the harvest to the re-harvest. However, the low light intensity condition affected the harvest yield. The first and second harvest yields, at 61 days and 96 days of treatment, are shown in Figure 4A,B, respectively. It was observed that the yield of *G. littoralis* was high in the light intensity of more than the 200 µmol∙m^−2^∙s^−1^ PPFD, where the harvest yield increased by more than twice as much during the second harvest compared to the first harvest in the 1500 µmol∙mol^−1^ CO_2_ concentration with 300 µmol∙m^−2^∙s^−1^ PPFD. However, regardless of the CO_2_ concentration, there was no significant difference between the first and second harvest yields in the 100 µmol∙m^−2^∙s^−1^ PPFD. This was because the high light intensity significantly affected the root development of *G. littoralis* and sufficient roots were produced for new leaf production. The change in the number of leaves from the harvest to the re-harvest is shown in Figure 5. The number of leaves was only affected by the light intensity, and in particular, leaves were developed the most in the 300 µmol∙m^−2^∙s^−1^ PPFD. Lee et al. [39] reported that new leaf emergence and biomass accumulation were promoted at a higher apparent daily light integral level. Furthermore, the higher light intensity produced more primary and total plant runners of strawberry.

### 2.5. Bioactive Compound

**Figure 6:**
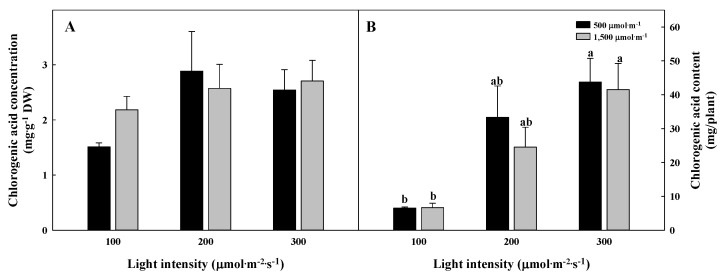
The different chlorogenic acid concentrations (**A**) and contents per biomass (**B**) of *Glehnia littoralis* Fr. Schmidt ex Miquel shoots, as affected by various CO_2_ concentrations and light intensities after 56 days of treatment. The vertical bars represent the standard deviation of the mean (*n* = 3). Different letters above bars indicate significant differences at *p* ≤ 0.05, using Tukey’s multiple range test.

**Figure 7:**
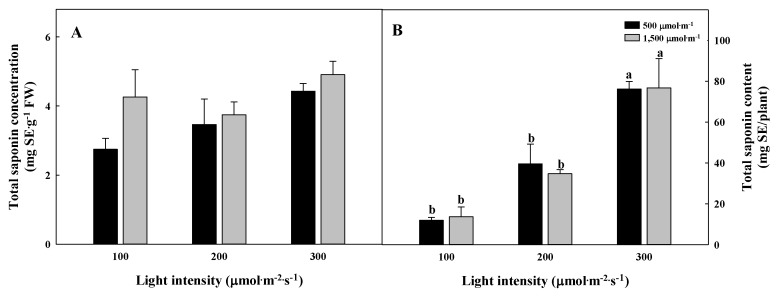
The different total saponin concentration (**A**) and content per biomass (**B**) of *Glehnia littoralis* Fr. Schmidt ex Miquel shoots, as affected by various CO_2_ concentrations and light intensities after 56 days of treatment. The vertical bars represent the standard deviation of the mean (*n* = 3). Different letters above bars indicate significant differences at *p* ≤ 0.05, using Tukey’s multiple range test.

The chlorogenic acid and total saponin concentrations per DW or FW of G. littoralis were not significantly different between the treatments (Figure 6A and Figure 7A). On the other hand, the chlorogenic acid and total saponin contents per biomass were the greatest in the 300 µmol∙m^−2^∙s^−1^ PPFD regardless of the CO_2_ concentration (Figure 6B and Figure 7B). In a CO_2_ concentration of 500 µmol∙mol^−1^, the chlorogenic acid and total saponin contents per biomass were higher in the 300 µmol∙m^−2^∙s^−1^ PPFD by 1.6 and 6.3 times compared to in the 100 µmol∙m^−2^∙s^−1^ PPFD, respectively. In other studies, CO_2_ enrichment improved the nutritional qualities but the total free phenolic acids and chicoric acid contents of lettuce significantly decreased; the reaction to the elevated CO_2_ concentration was found to be dependent on the plant species [40,41]. The accumulation of bioactive compounds is induced by the increased soluble sugar acting as a precursor that promotes the synthesis and accumulation of antioxidants [42,43,44]. The polyphenol content of lettuce grown in a 350 µmol∙m^−2^∙s^−1^ PPFD was significantly higher than that of plants grown in a 180 µmol∙m^−2^∙s^−1^ PPFD [45]. In this study, there was no difference in the concentration of total chlorogenic acid per DW or total saponin per FW, but the production of bioactive compounds increased in the 300 µmol∙m^−2^∙s^−1^ PPFD, which displayed high photosynthesis and superior growth. 

## 3. Materials and Methods

### 3.1. Plant Materials and Growth Condition

**Figure 8:**
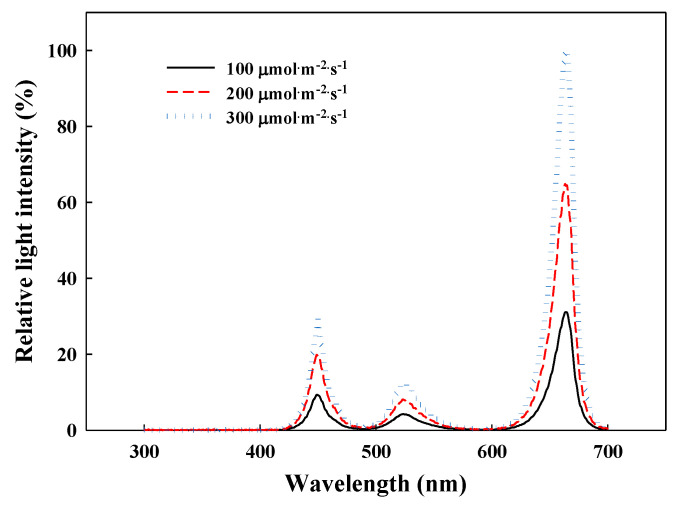
Relative spectral distribution of the RGB LEDs (red:green:blue = 7:1:2) used in a closed-type plant production system.

The G. *littoralis* seeds were sown in a Petri dish and the seedlings were placed in 128-hole plug trays filled with urethane sponge (Hydroponic Sponge, Easyhydro Co. Ltd., Chuncheon, Korea) in a CPPS (C1200H3, FC Poibe Co. Ltd., Seoul, Korea) at a temperature of 20 ± 1 °C, relative humidity (RH) of 60 ± 10%, a photoperiod of 12/12 h (light/dark), and a 150 µmol∙m^−2^∙s^−1^ PPFD using RGB (red:green:blue = 7:1:2) LEDs (ES LEDs Co. Ltd., Seoul, Korea). The 60-day-old seedlings were transplanted into a deep floating technique system with recycling Hoagland nutrient solution [46] in a CPPS. The plants were cultured for 96 days at a temperature of 20 ± 1 °C, RH of 60 ± 10%, and a photoperiod of 12/12 h (light/dark). The experimental treatments were set to 500 or 1500 µmol∙mol^−1^ CO_2_ concentrations in combination with one of three light intensities: 100, 200, or 300 µmol∙m^−2^∙s^−1^ PPFD, which were provided by RGB (red:green:blue = 7:1:2) LEDs (Figure 8). In CPPS where CO_2_ concentration, photoperiod, and temperature are automatically controlled, the CO_2_ concentration was controlled by connecting a liquefied carbon dioxide tank and CO_2_ regulator. The CO_2_ concentration, temperature, and RH were monitored during the cultivation period using a data logger (TR-76Ui, T&D Co. Ltd., Nagano, Japan). The light intensity was set using a photometer (HD2101.2, Delta Ohm SrL, Caselle, Italy).

### 3.2. Growth Characteristics

After 56 days of treatment, the petiole length, crown and root diameters, root length, FW and DW of the shoots and roots, number of leaves, and leaf area were measured. The FW was investigated using an electronic balance (EW220-3NM, Kern & Sohn GmbH., Balingen, Germany) and the DW was investigated after drying in an oven (Venticell-220, MMM Medcenter Einrichtunger GmbH., Planegg, Germany) at 70 °C for 72 h. The crown and root diameter were measured using Vernier calipers (CD-20CPX, Mitutoyo Co. Ltd., Kawasaki, Japan). The leaf area was measured using a leaf area meter (LI-3000, LI-COR Inc., Lincoln, NE, USA). Photosynthetic rate was measured using a portable photosynthesis system (CIRAS-3, PP Systems International Inc., Amesbury, MA, USA) on the fully unfolded fifth leaf from the top. The measurement conditions were controlled as follows: leaf area 4.5 mm^2^; leaf temperature 20 °C; air flow rate 150 mL·min^−1^; 500 or 1500 µmol∙mol^−1^ CO_2_ concentration; 100, 200, or 300 µmol∙m^−2^∙s^−1^ PPFD. The chlorophyll content was expressed as the SPAD, and measured using a portable chlorophyll meter (SPAD-502, Konica Minolta Inc., Tokyo, Japan). The first and second harvests were performed after treatment for 61 and 96 days, respectively, and the harvest yield was measured by weight of marketable leaves (over 15 cm^2^ of leaf area).

### 3.3. Total Sugar and Starch

For the total sugar content determination, the leaves of *G. littoralis* were ground with liquid nitrogen and stored in a deep freezer (NF-140SF, Nihon Freezer Co. Ltd., Yushima, Japan) at −70 °C. A 0.3 g sample was taken for each treatment. The samples were mixed with 10 mL of 80% ethanol and then ground for 1 min. After being heated in a 60 °C water bath, the supernatant was separated via centrifugation (908× *g*, 20 °C, 30 min). Then, 10 mL of 80% ethanol was added to the remaining precipitate, heated in a waterbath at 60 °C for 30 min, and centrifuged under the same conditions. After the two centrifugations, the supernatant was diluted with a total of 40 mL of 80% ethanol. Then, 0.5 mL of 5% phenol reagent was added to the sample solution, vortexed, and 2.5 mL of 98% sulfuric acid were added and vortexed. After cooling at room temperature, the absorbance was measured at 490 nm using a spectrophotometer and the total sugar content was calculated using glucose as the standard. 

For the starch determination, the leaves were ground with liquid nitrogen and stored in a deep freezer at −70 °C. Total of 0.3 g of sample was used for each treatment. To solubilize the sample, 10 mL of 80% ethanol was added, shaken for 30 min, and then centrifuged at 300× *g* for 30 min at 20 °C. Then, 40% ethanol was added to the residue, and centrifuged again under the same conditions. Then, 2 mL of 30% HClO_4_ and 1 mL of dimethyl sulfoxide were added to the residue and kept at room temperature for 30 min. Then, 2 mL of distilled water and 5 mL of H_2_SO_4_ were added, and the waterbath was used to maintain the temperature at 100 °C for 1 h, then centrigugation was performed under 300× *g* for 30 min at 20 °C. Around 0.5 mL of this sample solution was mixed with 0.5 mL of 5% phenol reagent, then vortexed, followed by the addition of 2.5 mL of 98% sulfuric acid and the vortexed. After cooling at room temperature, the absorbance was measured at 470 nm using a spectrophotometer, and the starch content was calculated using glucose as the standard. 

### 3.4. Bioactive Compounds

#### 3.4.1. Chlorogenic Acid Concentration

For the quantitative analysis of chlorogenic acid, 500 mg of each powdered plant material were first mixed with 20 mL of 80% methanol and then shaken at 100 rpm on an orbital shaker for 24 h at room temperature. Afterward, all of the supernatant was centrifuged at 4250× *g* for 5 min. The supernatant was filtered through a 0.2 μm syringe filter (25HP020AN, Advantech Co. Ltd., Asan, Korea) before being injected into a high-performance liquid chromatography device (Nexera, Shimadzu Corp., Kyoto, Japan) system equipped with an 4.6 × 150 mm, 5 μm column (Agilent Eclipse plus-C18, Agilent Technology Co Ltd., Santa Clara, CA, USA) and a guard column maintained at 30 °C. Solvents A (methanol) and B (trifluoroacetic acid) were used as the mobile phases. The gradient was as follows: 0 min, 100% A; 3 min, 10% B; 8 min, 30% B; 30 min, 50% B; 40 min, 60% B; 50 min, 100% B; held constant for 10 min. The flow rate was 0.8 mL·min−1 and the injection volume was 10 μL. The chromatogram was monitored at 270 nm using photodiode array detection. To present the chlorogenic acid, concentrations of the derivatized samples, standard curves were prepared using 3-(3,4-dihydroxycinnamoyl) quinic acid (chlorogenic acid, Sigma-Aldrich Co. Ltd., St. Louis, MO, USA). Then, the calculated values were converted to the concentrations in terms of milligrams of chlorogenic acid, per grams of DW of the samples.

#### 3.4.2. Total Saponin Concentration 

To measure the total saponin concentration of the root, the total saponin content was extracted using a method modified from [47]. About 0.5 g root powder was defatted with 10 mL of petroleum ether by shaking it for 4 h, and then the residues were extracted twice, each with 5 mL of 80% aqueous methanol, by shaking for 4 h each time on an orbit shaker. The extracts were stored at 4 °C in the dark for later use. Approximately 100 μL of the extract was mixed with 400 μL of 80% methanol, 500 μL of 8% vanillin solution, and 5 mL of 72% sulfuric acid. After the mixture was heated in a water bath at 60 °C for 10 min, it was cooled in ice-cold water. The absorbance of the supernatant was measured using a spectrophotometer at 544 nm to determine the total saponin concentration, which was expressed as milligrams of saponin equivalent (SE) per grams of fresh weight.

### 3.5. Statistical Analysis

The experiment involved three replicates and ten plants per replicate, and was laid out in a completely randomized block design. After selecting uniform plants, three plants per replicate were used to determine the plant growth parameters and three plants per replicate were used to determine the photosynthetic rate; the total sugar, starch, and bioactive compounds; and the harvest yield. The statistical analyses were carried out using the SAS program (SAS 9.4, SAS Institute Inc., Cary, NC, USA). The experimental results were subjected to an analysis of variance (ANOVA) and Tukey’s multiple range tests. Graphing was performed with the SigmaPlot program (SigmaPlot 12.0, Systat Software Inc., Palo Alto, CA, USA).

## 4. Conclusions

This study focused on the effects of CO_2_ concentration (500 or 1500 µmol∙mol^−1^) and light intensity (100, 200, or 300 µmol∙m^−2^∙s^−1^ PPFD) on the growth, photosynthetic rate, and bioactive compound content of *G. littoralis* to find an appropriate CO_2_ concentration and light intensity for the high-quality, mass production of medicinal plants grown in a CPPS. The *G. littoralis* was not affected by the CO_2_ concentration, while a high light intensity increased the growth, bioactive compound content, and harvest yield. The data showed that a 300 µmol∙m^−2^∙s^−1^ PPFD greatly enhanced the plant production in a CPPS.

## Figures and Tables

**Table 1:** The different shoot growth characteristics of *Glehnia littoralis* Fr. Schmidt ex Miquel, as affected by various CO_2_ concentrations and light intensities after 56 days of treatment (*n* = 3).

CO_2_ Concentration (µmol∙mol^−1^) (A)	Light Intensity (µmol∙m^−2^∙s^−1^) (B)	Petiole Length (cm)	Leaf Area (cm^2^/plant)	Crown Diameter (mm)	No. of Leaves	Fresh Weight (g/plant)	Dry Weight (g/plant)
500	100	8.8 ± 0.9 a ^z^	82.65 ± 13.1 bc	8.0 ± 0.6 bc	8.0 ± 0.6 bc	5.93 ± 1.1 bc	1.28 ± 0.2 bc
	200	7.0 ± 0.7 ab	112.23 ± 19.7 ab	13.5 ± 2.1 ab	12.2 ± 2.1 ab	8.19 ± 1.7 ab	1.61 ± 0.4 ab
	300	5.3 ± 0.4 b	142.78 ± 13.4 a	13.8 ± 1.7 a	14.2 ± 3.0 ab	10.85 ± 0.7 a	2.17 ± 0.2 a
1500	100	6.6 ± 0.7 ab	46.60 ± 9.5 c	7.6 ± 0.5 c	6.8 ± 1.4 c	3.12 ± 0.6 c	0.58 ± 0.1 c
	200	5.0 ± 0.3 b	104.33 ± 15.1 ab	14.7 ± 1.4 a	16.5 ± 1.2 a	7.55 ± 1.2 ab	1.53 ± 0.2 ab
	300	5.2 ± 0.2 b	107.04 ± 17.7 ab	13.8 ± 0.6 a	12.7 ± 1.4 ab	9.15 ± 1.1 ab	2.01 ± 0.2 ab
Significance ^y^	A	**	*	NS	NS	NS	NS
	B	***	**	***	***	***	***
	A × B	NS	NS	NS	NS	NS	NS

^z^ Mean separation within columns using Tukey’s multiple range test at *p* ≤ 0.05. ^y^ NS, *, **, *** Nonsignificant or significant at *p* ≤ 0.05, 0.01, or 0.001, respectively.

**Table 2:** The different root growth characteristics of *Glehnia littoralis* Fr. Schmidt ex Miquel, as affected by various CO_2_ concentrations and light intensities after 56 days of treatment (*n* = 3).

CO_2_ Concentration (µmol∙mol^−1^) (A)	Light Intensity (µmol∙m^−2^∙s^−1^) (B)	Root Length (cm)	Root Diameter (mm)	Fresh Weight (g/plant)	Dry Weight (g/plant)
500	100	10.7 ± 0.4 b ^z^	9.6 ± 0.5 ab	4.53 ± 0.2 cd	1.20 ± 0.1 c
	200	29.9 ± 1.5 a	11.0 ± 0.3 a	11.60 ± 2.4 ab	1.68 ± 0.4 bc
	300	26.3 ± 2.2 a	12.0 ± 0.7 a	16.60 ± 0.7 a	2.71 ± 0.1 a
1500	100	11.3 ± 0.5 b	6.5 ± 0.8 b	3.81 ± 0.7 d	0.80 ± 0.2 c
	200	25.5 ± 1.6 a	10.4 ± 1.3 a	10.12 ± 1.3 bc	1.51 ± 0.4 bc
	300	31.7 ± 1.5 a	12.3 ± 0.4 a	14.83 ± 1.8 ab	2.20 ± 0.2 ab
Significance ^y^	A	NS	NS	NS	NS
	B	***	***	***	***
	A × B	**	NS	NS	NS

^z^ Mean separation within columns using Tukey’s multiple range test at *p* ≤ 0.05. ^y^ NS, **, *** Nonsignificant or significant at *p* ≤ 0.01 or 0.001, respectively.
